# Boerhaave syndrome following the use of Kambo from the Amazonian *Phyllomedusa bicolor tree* frog in Australia

**DOI:** 10.1093/omcr/omad036

**Published:** 2023-04-20

**Authors:** Christopher R Darlington, Nicolas Copertino

**Affiliations:** Department of General Surgery, Sunshine Coast University Hospital, Birtinya, Queensland 4575, Australia; Department of General Surgery, Sunshine Coast University Hospital, Birtinya, Queensland 4575, Australia

## Abstract

This case report describes Boerhaave syndrome in an otherwise healthy male after Kambo use in Australia. Kambo is produced from the sections of the *Phyllomedusa bicolor* (Boddaert, 1772) *tree frog.* Also known as the giant leaf frog, two-coloured leaf frog, waxy-monkey tree frog and kambô, is native to South America. Its use is part of a traditional cleansing ceremony but has been adopted by the alternative health movement.

## INTRODUCTION

The rise of the global alternative health movement has seen the spread of many cultural rituals far from their origins [1]. This is problematic as people may undertake such practices unaware of possible risks, steps in preparation or traditional dosages. One such practice is the use of Kambo as a cleansing ritual. Once localized to South America, it is now practiced internationally [2].

## CASE DESCRIPTION

**Figure 1:**
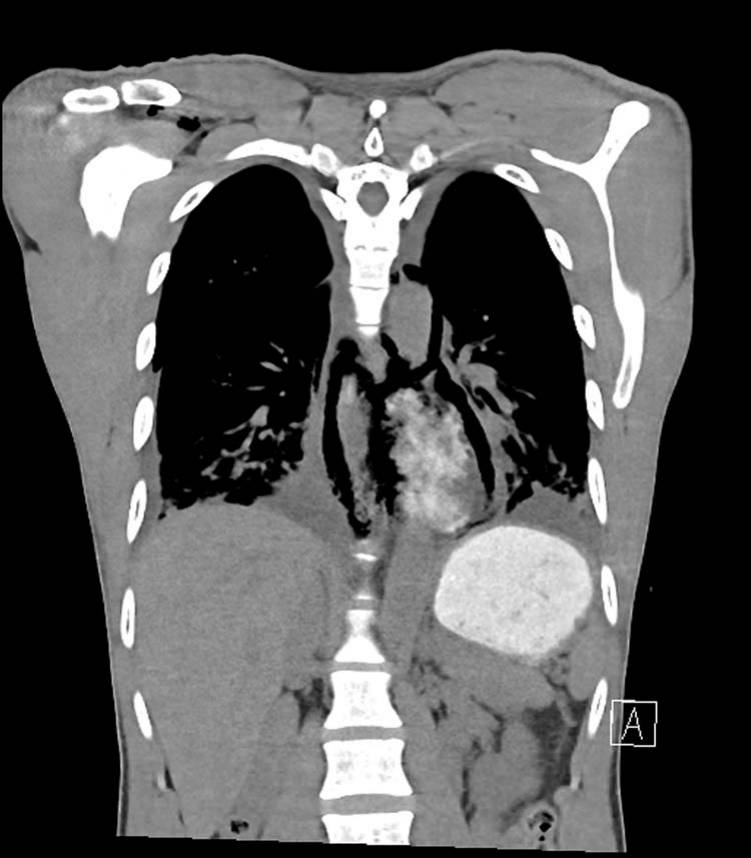
Large posterior mediastinal fluid and gas collection containing contrast indicating Boerhaave.

**Figure 2:**
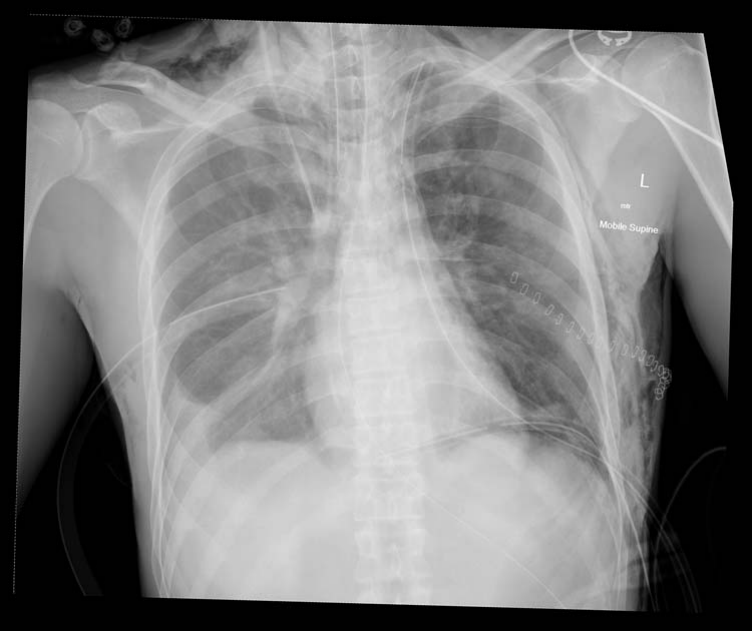
Post operative chest X-ray with bilateral chest drains and residual subcutaneous emphysema.

A 37-year-old male presented to the Emergency Department at Sunshine Coast University Hospital (Queensland, Australia) with severe chest and upper abdominal pain associated with protracted vomiting. This occurred after participating in a Kambo demonstration. Initial observations were normal (white cell count, 13.9 × 10^9^/L), however, over the next 2 h he demonstrated signs of septic shock. Clinical assessment indicated subcutaneous emphysema of the neck. Concern for Boerhaave syndrome was raised, broad spectrum antibiotic including antifungal therapy was commenced, and a computerized tomography (CT) scan with oral contrast was ordered. The CT scan revealed a perforation at the left lateral aspect of the distal oesophagus with extravasation of contrast into a large posterior mediastinal collection (Fig. 1). Urgent operative intervention involved a left posterolateral thoracotomy, revealing a defect in the distal oesophagus and a large mediastinal collection. The defect was closed with interrupted absorbable sutures and several large bore chest drains were placed (Fig. 2). A concomitant venting gastrostomy and feeding jejunostomy were placed to aid in early commencement of enteral nutrition. A contrast swallow at day 5 showed no ongoing leak, after which an oral diet was gradually recommenced. The patient was discharged 18 days post presentation. A follow-up endoscopy was performed at 3 months, which demonstrated a healed oesophagus.

## DISCUSSION

Boerhaave Syndrome is an uncommon and potentially fatal condition [3]. The pathophysiology is forceful emesis with sudden elevation of intra-oesophageal pressures resulting in rupture of the oesophagus. In most cases there is no underlying abnormality to the oesophagus, however risk factors include alcoholism and binge eating [4]. A classic triad (Macklers) has been described (chest pain, vomiting and subcutaneous emphysema), but is only present in 27% of presentations so a high degree of clinical suspicion is required [5, 6]. Patient outcome is strongly dependent on time to diagnosis, as well as perforation site. A 2010 study based on 27 patients across the 2002–2006 period, showed that perforations treated within 24 h showed a 6% mortality rate, compared with a 40% mortality rate in those diagnosed after 24 h [3].

This is the second published case of Boerhaave’s Syndrome due to Kambo use, and the first reported in Australia. In 2020, a 62-year-old woman in North America presenting with chest pain also demonstrated a rupture requiring surgical intervention [7]. Kambo originates from South America and is used as part of a cleansing ceremony. It is derived from secretions collected from the frog, *Phyllomedusa bicolor* and has been found to contain several active peptides including phyllocaeruleins (hypotensive properties), tachykinins and phyllokinins (vasodilators), dermorphins and deltorphins (opiate like actions) and adenoregulins (antiboiotics properties) [2]. The ritual describes drinking a large volume of water then applying Kambo to previously created burns, typically on the arm or thigh. Other complications of use include hepatitis, hyponatraemia and psychosis [8–10].

**Figure 3:**
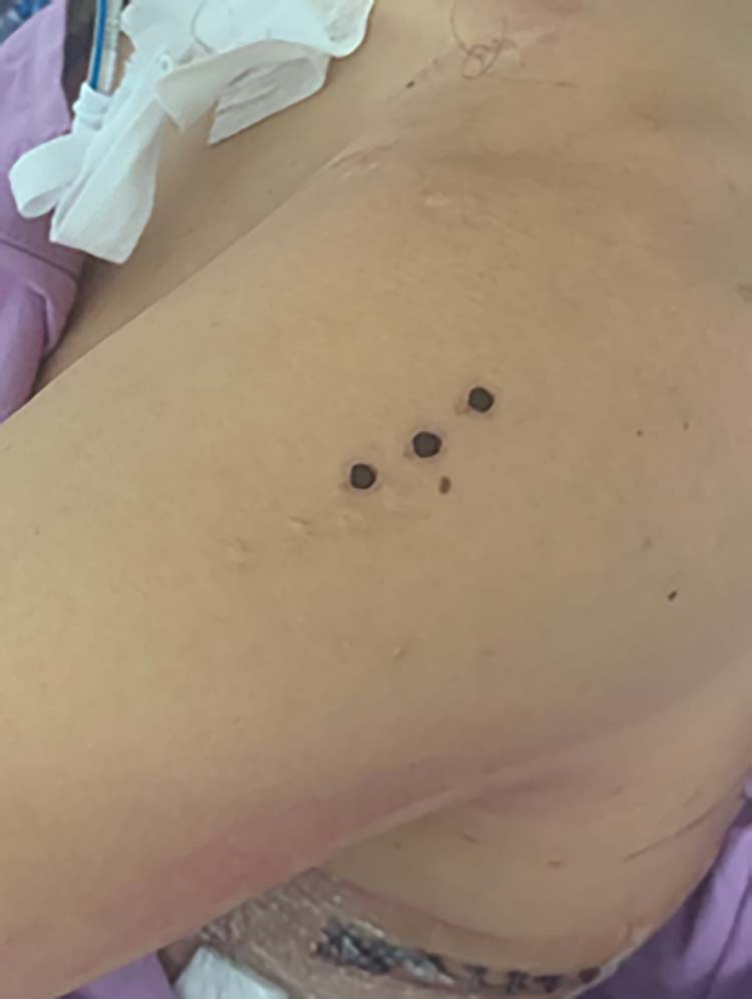
Left deltoid Kambo administration site. Black dots site of Kambo application (Taken with patient permission).

Our patient stated this was the second time he participated in this ritual. Whilst the first was out of curiosity, this occasion was part of a demonstration at a local centre. Both new (dark dots) and old scars are visible in Fig. 3. He stated the material had been sourced from an international group promoting Kambo use.

As traditional medicines enter international markets complications of their use can be expected far from where the practices originate. This is demonstrated by this instance of an oesophageal perforation in Australia, resulting as a complication of a traditional Amazonia practice.

## Data Availability

The data supporting this case report is available from the corresponding author on reasonable request.
